# Author Correction: NFAT primes the human *RORC* locus for RORγt expression in CD4+T cells

**DOI:** 10.1038/s41467-019-13451-4

**Published:** 2019-11-26

**Authors:** Hanane Yahia-Cherbal, Magda Rybczynska, Domenica Lovecchio, Tharshana Stephen, Chloé Lescale, Katarzyna Placek, Jérome Larghero, Lars Rogge, Elisabetta Bianchi

**Affiliations:** 1 Institut Pasteur, Immunoregulation Unit, Department of Immunology, Paris, France; 2 Université Paris Diderot, Sorbonne Paris Cité, Cellule Pasteur, Paris, France; 3 Institut Pasteur, Unité de Technologie et Service Cytométrie et Biomarqueurs (UTechS CB), Centre de recherche translationnelle (CRT), Paris, France; 4 Institut Pasteur, Genome Integrity, Immunity and Cancer Unit, Equipe Labellisée Ligue Contre le Cancer, Department of Immunology, Department of Genomes and Genetics, Paris, France; 5 Assistance Publique-Hopitaux de Paris, Hôpital Saint-Louis, Cell Therapy Unit and Cord Blood Bank; CIC de Biothérapies, CBT501 Paris, France; 6 Present Address: Laboratoire Colloides et Matériaux Divisés, École supérieure de Physique et de Chimie industrielles, Paris, France; 7 Present Address: Immunology and Metabolism, LIMES Institute, University of Bonn, Bonn, Germany

**Keywords:** Adaptive immunity, Gene regulation in immune cells, T-helper 17 cells, NF-kappaB

Correction to: *Nature Communications* 10.1038/s41467-019-12680-x, published online 16 October 2019.

The original version of this Article contained an error in the author affiliations.

Affiliation 4 incorrectly read ‘Institut Pasteur, Departments of Immunology and Genomes and Genetics, Paris, France’. The correct affiliation 4 should read as ‘Institut Pasteur, Genome Integrity, Immunity and Cancer Unit, Equipe Labellisée Ligue Contre le Cancer, Department of Immunology, Department of Genomes and Genetics, Paris, France’.

This has now been corrected in both the PDF and HTML versions of the Article.

