# Supplementary material for: NFAT primes the human RORC locus for RORγt expression in CD4+ T cells
Source: Nat Commun. 2019 Oct 16;10:4698. doi: 10.1038/s41467-019-12680-x (PMC6795897; doi:10.1038/s41467-019-12680-x)
Supplement: Supplementary file 1 — Supplementary Information [file 41467_2019_12680_MOESM1_ESM.pdf]

Supplementary Information for

NFAT primes the human *RORC* locus for RORγt expression in CD4+ T cells

Hanane Yahia-Cherbal et al.

This PDF file includes:
Supplementary Figures 1-7
Supplementary Tables 1-8

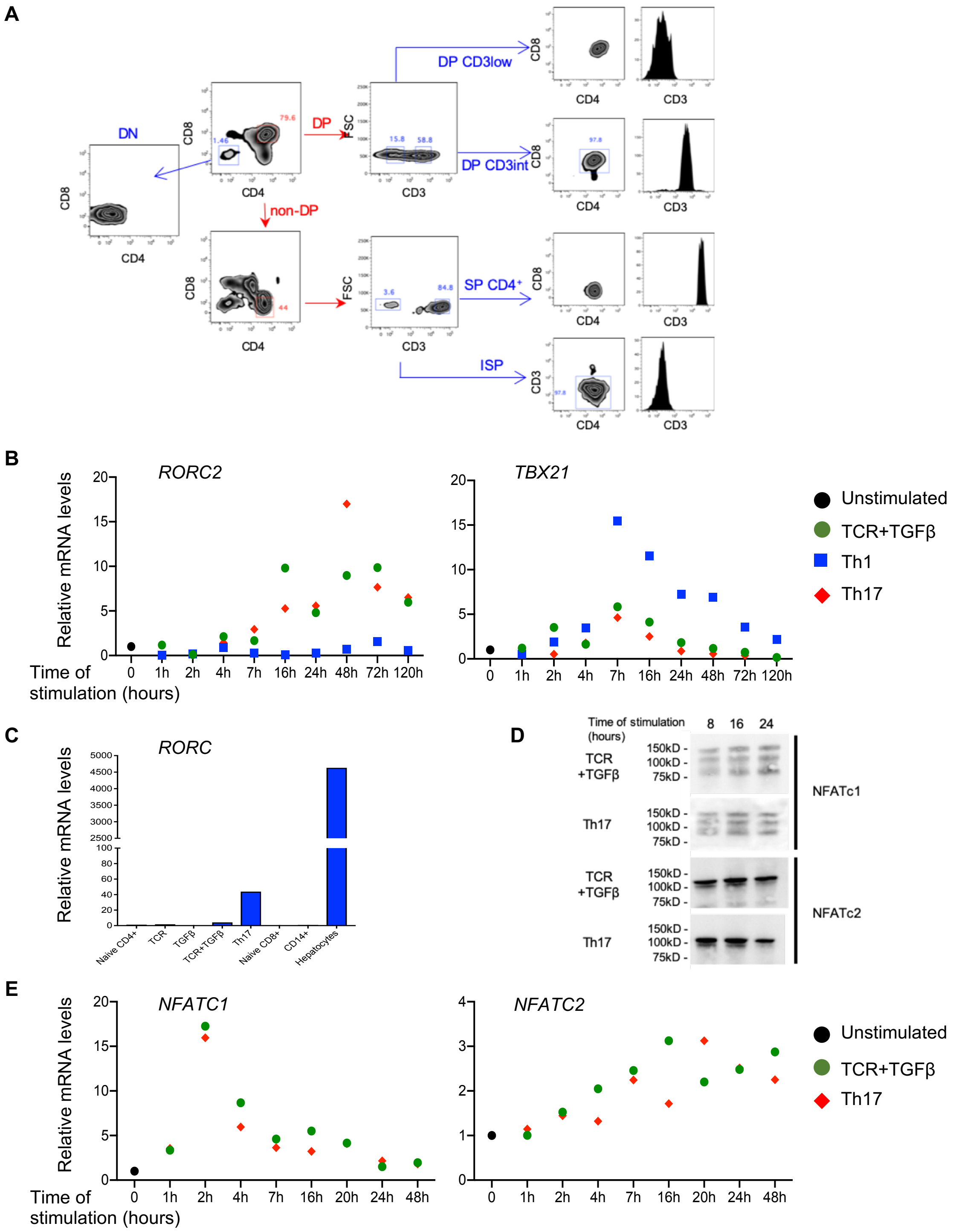

SUPPLEMENTARY FIGURE 1. *RORC2, RORC, TBX21, NFATc1 and NFATc2* expression in stimulated cord blood CD4$^+$ T cells. A. Gating strategy for thymic cell populations sorting. **B.** Time course of *RORC2* and Tbet (*TBX21*) mRNA expression. Cord blood CD4$^+$ T lymphocytes were stimulated in TCR+TGFb, Th1 or Th17 conditions for the times shown on the x-axis. Relative mRNA levels of the indicated genes were quantified by RT-qPCR, normalized to *18S*, and plotted relative to the unstimulated sample (one representative experiment of 6). **C.** *RORC* mRNA expression in cord blood CD4$^+$ T cells stimulated for 20 hours in different conditions. Naïve CD8$^+$ cells, CD14$^+$ cells (sorted from cord blood) and human hepatocytes were also analysed (one representative experiment of 2). **D.** NFATc1 and NFATc2 proteins in stimulated cord blood CD4$^+$ T cells. CD4$^+$ T lymphocytes were isolated from cord blood and stimulated for the indicated time points. The western blot of the cell lysates was probed with anti-NFATc1 or anti-NFATc2 antibodies (one representative experiment of 4) **E.** Time course of *NFATc1* and *NFATc2* expression in stimulated cord blood CD4$^+$ T cells. The mRNA levels were quantified by RT-qPCR, normalized to *18S* and plotted relative to the levels in the unstimulated sample (one representative experiment of 4). *Source data are provided as a Source Data file.*

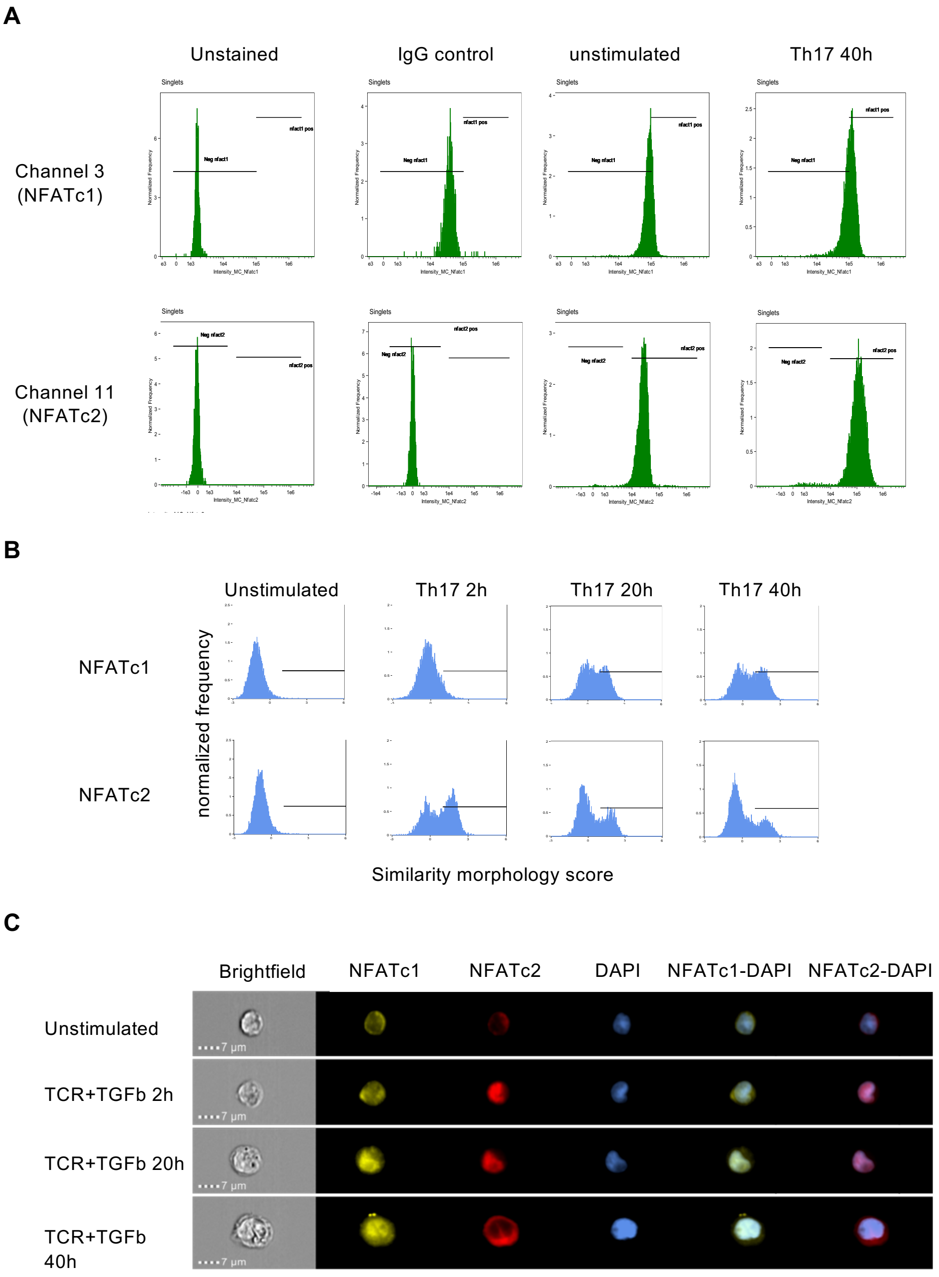

SUPPLEMENTARY FIGURE 2 A-C. Imaging Flow Cytometry analysis of NFAT nuclear translocation in cord blood derived CD4$^+$ T cells. A. Negative and positive gates were set on DAPI-positive, single cells stained with an irrelevant IgG control, as indicated by the horizontal bars. **B.** Nuclear translocation was computed as a "similarity score" (x-axis), which is based on a regression analysis of pairs of intensity values taken from the NFAT and DAPI fluorescence images of single cells in focus. **C.** Representative images of cord blood derived CD4$^+$ T cells stimulated through the TCR in the presence of TGFb for the indicated times. The columns show, respectively, the pseudo-colored images for NFATc1, NFATc2, DAPI staining, and the overlay of NFATc1 with DAPI, or NFATc2 with DAPI. *Raw image files are available from the authors upon request.*

D

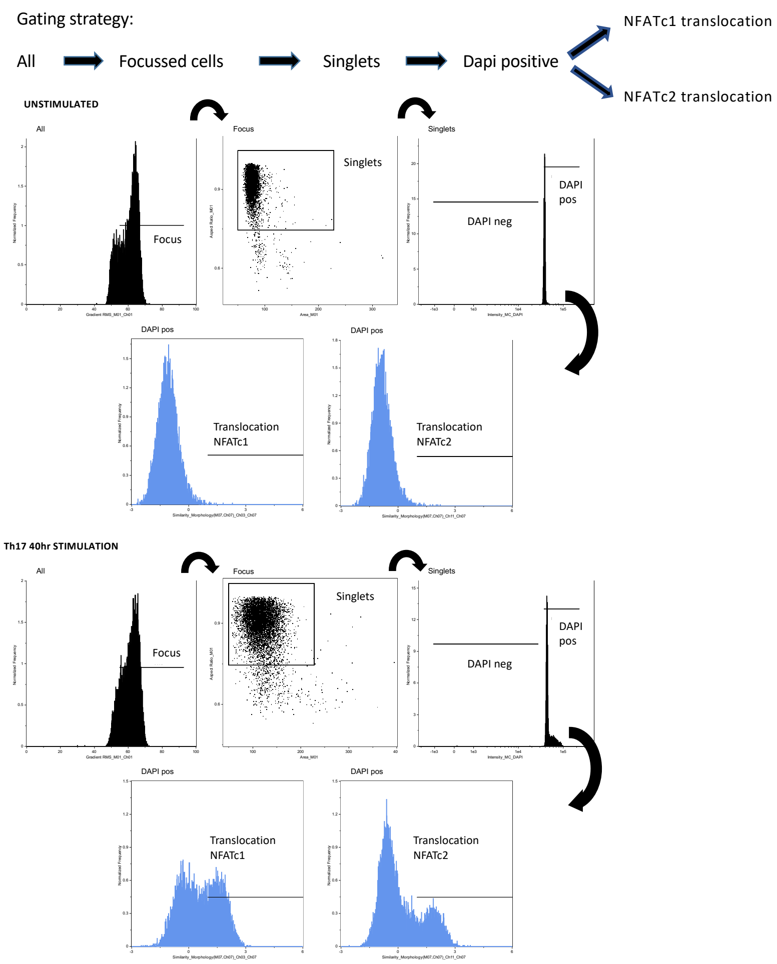

SUPPLEMENTARY FIGURE 2 D. Gating Strategy for ImageStream analysis

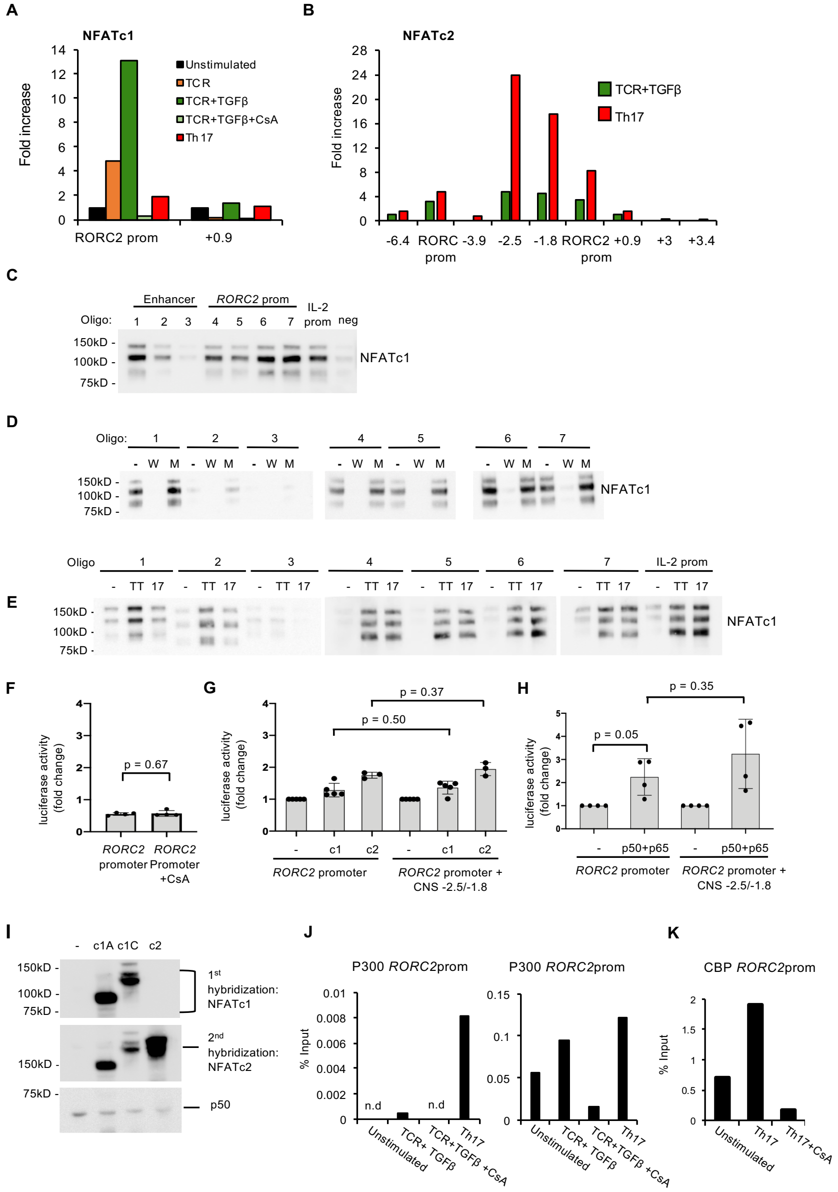

SUPPLEMENTARY FIGURE 3. NFAT proteins bind to the *RORC* locus. ChIP was performed with an anti-NFATc1 antibody in **A** or an anti-NFATc2 antibody in **B** on cord blood $CD4^+$ T lymphocytes, cultured for 20 hours. Shown is one representative experiment of three **C.** NFATc1 binds to several sites in the *RORC* locus *in vitro*. DNA affinity capture was performed on lysates from Jurkat E6.1 cells stimulated with PMA/ionomycin for 30 minutes. **D.** DNA affinity competition capture assay (see figure 5D). Western blot of the oligo-bound proteins was probed with anti-NFATc1 antibodies. **E.** DNA affinity capture assay on lysates from cord blood $CD4^+$ T cells stimulated in the indicated conditions ("-": unstimulated, TT: TCR+TGFβ; 17: Th17 polarizing condition). **F.** NFAT-independent basal *RORC2* promoter activity. HEK293T cells were transfected with the *RORC2* promoter reporter constructs in the presence or absence cyclosporinA. Luciferase activity in the lysates was measured after 20h. Average and SD from 5 transfections. **G** and **H.** The *RORC2* CNS-2.5/-1.8 region does not further increase transcriptional activation of the *RORC2* promoter *in vitro*. HEK293T cells were transfected with reporter constructs containing the *RORC2* promoter, or the *RORC2* promoter plus the CNS-2.5/-1.8 region, in the presence or absence of the indicated proteins (c1: NFATc1; c2: NFATc2. Luciferase activity in the lysates was measured after 20h. Average fold increase relative to the *RORC2* promoter alone and SD from 5 (G) or 4 (H) transfections. Shown are p-values from two-tailed t-tests between the indicated conditions. **I.** Western blot of ectopically expressed NFAT proteins in HEK293T cell lysates (c1A: NFATC1A; c1C: NFATc1C and c2: NFATc2). **J**, **K.** P300 and CBP RT-qPCR-ChIPs were performed in peripheral blood $CD3^+$ T cells stimulated for 20 hours in the shown conditions. n.d: not detected. Shown is the average of 3 technical replicates of one representative experiment of 4 (p300) and 3 (CBP). *Source data are provided as a Source Data file.*

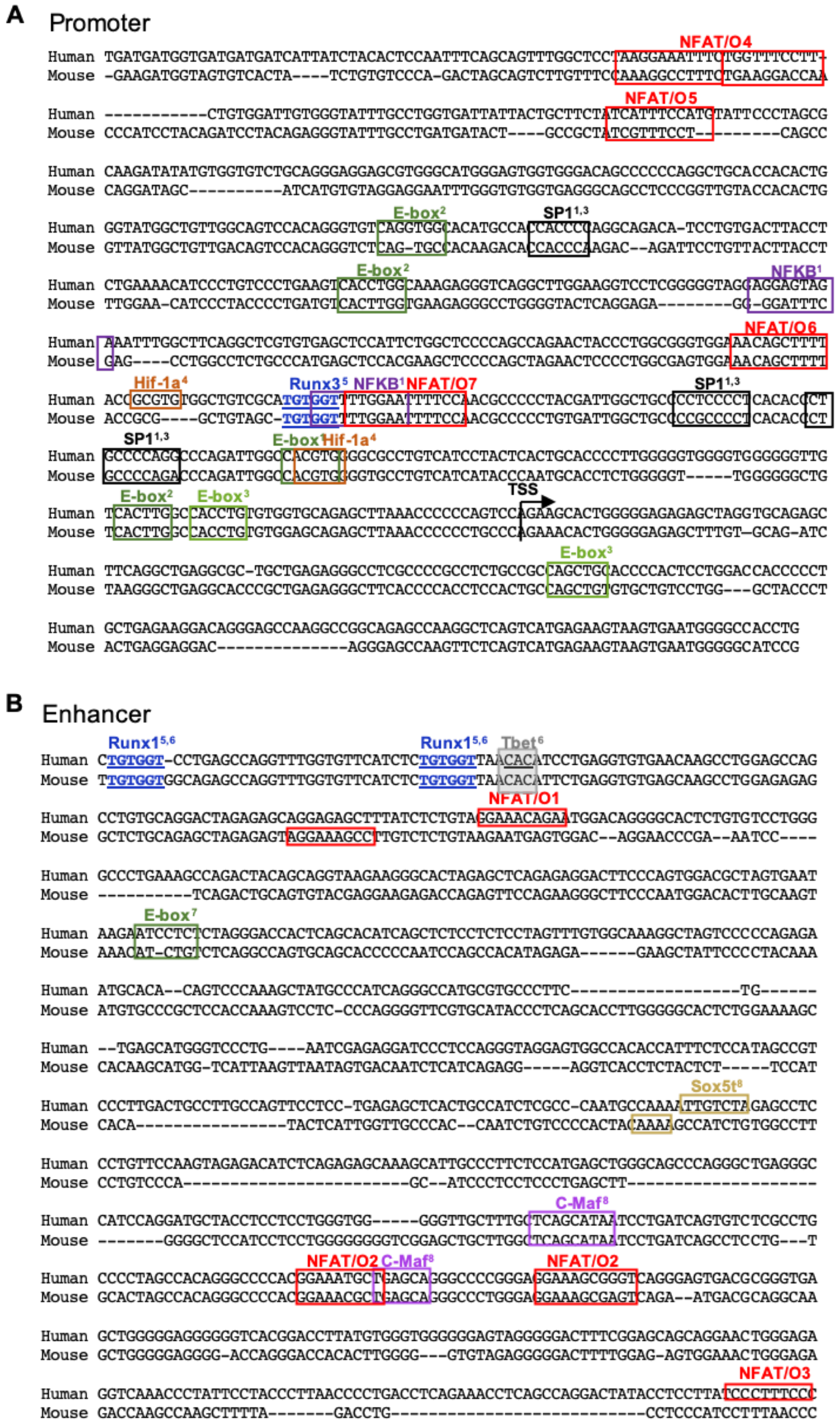

SUPPLEMENTARY FIGURE 4. Sequence alignment of the human and mouse RORC regulatory regions. A. Sequence alignment of the human and mouse *RORC2* promoter region. The major Transcription Start Site (TSS) identified by RACE in mouse Th17 cells is indicated by an arrow(1). **B.** Sequence alignment of the human and mouse *RORC* CNS-2.5/-1.8 regulatory region. NFAT binding sites characterized in this study are squared in red. Also indicated are binding sites for transcription factors previously described to regulate murine[1,3-8] or human[2] RORgt expression (see references below):

(1)Ruan, Q., Kameswaran, V., Zhang, Y. *et al.* 2011. The Th17 Immune Response Is Controlled by the Rel-RORγ-RORγ T Transcriptional Axis. *J. Exp. Med.* 208 (11): 2321–33. (2)Ratajewski, M., A. Walczak-Drzewiecka, A. Salkowska, *et al*. 2012. Upstream Stimulating Factors Regulate the Expression of RORγT in Human Lymphocytes. *J. Immunol.* 189 (6): 3034–42. (3)Xi, H., Schwartz, R., Engel, I. *et al.* 2006. Interplay between RORγt, Egr3, and E Proteins Controls Proliferation in Response to Pre-TCR Signals. *Immunity* 24 (6): 813–26.
(4)Dang, E. V., Barbi, J., Yang, H.-Y., *et al*. 2011. Control of TH17/Treg Balance by Hypoxia-Inducible Factor 1. *Cell* 146 (5): 772–84.
(5)Liu, H.-P., Cao, A. T., Feng, T. *et al.* 2015. TGF-β Converts Th1 Cells into Th17 Cells through Stimulation of Runx1 Expression. *Eur. J. Immunol*. 45 (4): 1010–18.
(6)Lazarevic, V., Chen, X., Shim, J.-H., *et al.* 2011. T-Bet Represses T(H)17 Differentiation by Preventing Runx1-Mediated Activation of the Gene Encoding RORγt. *Nat. Immunol*. 12 (1): 96–104.
(7)Zhang, F., Fuss, I. J., Yang, Z. *et al*. 2014. Transcription of RORγt in Developing Th17 Cells Is Regulated by E-Proteins. *Mucosal Immunol* 7 (3): 521–32.
(8)Tanaka, S., Suto, A., Iwamoto, T., *et al.* 2014. Sox5 and C-Maf Cooperatively Induce Th17 Cell Differentiation via RORγt Induction as Downstream Targets of Stat3. *J. Exp. Med.* 211 (9): 1857–74.
NFATc2 binding to the promoter region (exact site not characterized): Kyun-Do K., Sonal S., Yossan-Var T., *et al.*2014. Calcium Signaling via Orai1 Is Essential for Induction of the Nuclear Orphan Receptor Pathway To Drive Th17 Differentiation. Journal of Immunology. 192: 110-122.

A

Promoter

Upstream O4
ATCCACCCACCTTGGGCTCCCAAAGTGCTGGGATTACAGGCGTGAGCCACTGCCCCCGGCCCACATAAAGTATTTTGT

GAGGATTAGATTATCCACATAAAACATCGAGAACCATTTCTGACACATAATAGTAAGCACTATTATTATGATTATGAGT

NFAT/O4
ATGATGATGGTGATGATGATCATTATCTACACTCCAATTTCAGCAGTTTGGCTCCTAAGGAAATTTCTGGTTTCCTT

NFAT/O5
CTGTGGATTGTGGGTATTTGCCTGGTGATTATTACTGCTTCTATCATTTCCATGTATTCCCTAGCGCAAGATATATGTG

Downstream O5
GTGTCTGCAGGGAGGAGCGTGGGCATGGGAGTGGTGGGACAGCCCCCCAGGCTGCACCACACTGGGTATGGCTGTTGGC

AGTCCACAGGGTGTCAGGTGGCACATGCCACCACCCCAGGCAGACATCCTGTGACTTACCTCTGAAAACATCCCTGTCC

Upstream O6
CTGAAGTCACCTGGCAAAGAGGGTCAGGCTTGGAAGGTCCTCGGGGGTAGGAGGAGTAGAAATTTGGCTTCAGGCTCGT

NFAT/O6
GTGAGCTCCATTCTGGCTCCCCAGCCAGAACTACCCTGGCGGGTGGAAACAGCTTTTACCGCGTGTGGCTGTCG

NFAT/O7
CATGTGGTTTTGGAATTTTCCAACGCCCCCTACGATTGGCTGCCCCTCCCCTCACACCCTGCCCCAGGCCCAGATTGGC

Downstream O7
CACGTGGGGCGCCTGTCATCCTACTCACTGCACCCCTTGGGGGTGGGGTGGGGGGTTGTCACTTGGCCACCTGTGTGGT

TSS
GCAGAGCTTAAACCCCCCAGTCCAGAAGCACTGGGGGAGAGAGCTAGGTGCAGAGCTTCAGGCTGAGGCGCTGCTGAGA

GGGCCTCGCCCCGCCTCTGCCGCCAGCTGCACCCCACTCCTGGACCACCCCCTGCTGAGAAGGACAGGGAGCCAAGGCC

GGCAGAGCCAAGGCTCAGTCATGAGAAGTAAGTGAATGGGGCCACCTGGGGGCGGGGGAGCCTGGACCCTGTCG

B

Enhancer

Upstream O1
CTGTGGTCCTGAGCCAGGTTTGGTGTTCATCTCTGTGGTTAACACATCCTGAGGTGTGAACAAGCCTGGAGCCAG

NFAT/O1
CCTGTGCAGGACTAGAGAGCAGGAGAGCTTTATCTCTGTAGGAAACAGAATGGACAGGGGCACTCTGTGTCCTGG

GGCCCTGAAAGCCAGACTACAGCAGGTAAGAAGGGCACTAGAGCTCAGAGAGGACTTCCCAGTGGACGCTAGTGA

Downstream O1
ATAAGAATCCTCTCTAGGGACCACTCAGCACATCAGCTCTCCTCTCCTAGTTTGTGGCAAAGGCTAGTCCCCCAG

AGAATGCACACAGTCCCAAAGCTATGCCCATCAGGGCCATGCGTGCCCTTCTGTGAGCATGGGTCCCTGAATCGA

GAGGATCCCTCCAGGGTAGGAGTGGCCACACCATTTCTCCATAGCCGTCCCTTGACTGCCTTGCCAGTTCCTCCT

GAGAGCTCACTGCCATCTCGCCCAATGCCAAAATTGTCTAGAGCCTCCCTGTTCCAAGTAGAGACATCTCAGAGA

Upstream O2
GCAAAGCATTGCCCTTCTCCATGAGCTGGGCAGCCCAGGGCTGAGGGCCATCCAGGATGCTACCTCCTCCTGGGT

NFAT/O2
GGGGGTTGCTTTGCTCAGCATAATCCTGATCAGTGTCTCGCCTGCCCCTAGCCACAGGGCCCCACGGAAATGCTG

NFAT/O2
AGCAGGGCCCCGGGAGGAAAGCGGGTCAGGGAGTGACGCGGGTGAGCTGGGGGAGGGGGGTCACGGACCTTATGT

GGGTGGGGGGAGTAGGGGGACTTTCGGAGCAGCAGGAACTGGGAGAGGTCAAACCCTATTCCTACCCTTAACCCC

Downstream O2
TGACCTCAGAAACCTCAGCCAGGACTATACCTCCTTATCCCTTTCCCATCCCACCAGTCCTCGTGTCCCCACCCC

AAGAGTGTTGTCCTGAGGATCCAGGCTGACAGCCTCTCCCCCACTCCACGGCCCAGCTCCTGAAGCCCCAGGAAG

GCCCAGGAAGGCAAGGAAAGGCCTGTGCCTGGATGAGAGGGTCTGGAGTCCCAGCAAGATCAGACACCTCTCAGG

SUPPLEMENTARY FIGURE 5. Position of crRNA used for CRISPR/Cas9-directed deletions at the human *RORC* regulatory regions. A. Human sequence of the *RORC2* promoter region. The major Transcription Start Site (TSS) is indicated by an arrow. **B.** Human *RORC* CNS-2.5/-1.8 regulatory region. NFAT binding sites characterized in this study are squared in red. Also indicated are the crRNA used in the CRISPR/Cas9 experiment. “Upstream” and “Downstream” oligos were paired to obtain deletion of the intervening sequence.

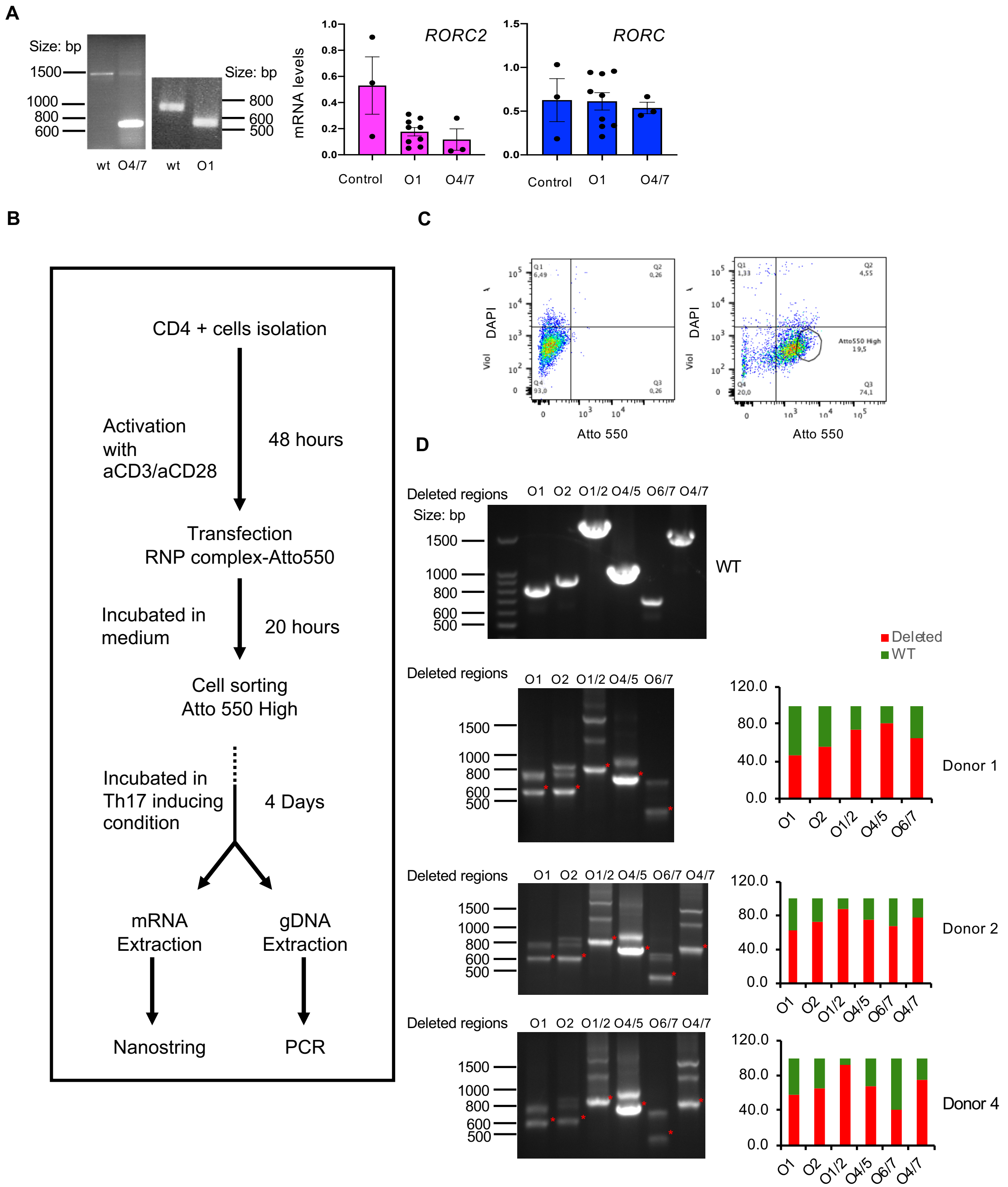

SUPPLEMENTARY FIGURE 6. CRISPR/Cas9-dependent deletions of regulatory regions at the *RORC2* locus. A. Analysis of Jurkat clones carrying CRISPR/Cas9-mediated deletions of the enhancer region O1 and the promoter region O4/7. Left panels: PCR amplification of the targeted regions in negative control Jurkat clones (wt) and in the deleted clones. Right panels: average and SD of *RORC* and *RORC2* mRNA levels in 3 to 9 clones. **B.** Workflow chart of CRISPR/Cas9 experiments in primary CD4^{+} T cells. **C.** Sorting strategy of CRISPR/Cas9 transfected CD4^{+} T cells. Left: CD4^{+} T cells incubated with the ribonuclear protein (RNP) complex but not electroporated were used to set the gate for positive cells. Right: O4/7 deleted cells. Shown is the sorting gate for the CRISPR/Cas9 enriched cells (Atto550 High, 15-20% of Atto550 positive cells). **D.** PCR amplification of genomic DNA from untransfected CD4^{+} T cells ("wild type, WT"), and from sorted CRISPR/Cas9 transfected samples (donor 1, donor 2, donor 4). The asterisk indicates the band carrying the expected deletion. Quantification of the PCR bands are shown on the right panels (ImageJ software). *Source data are provided as a Source data file.*

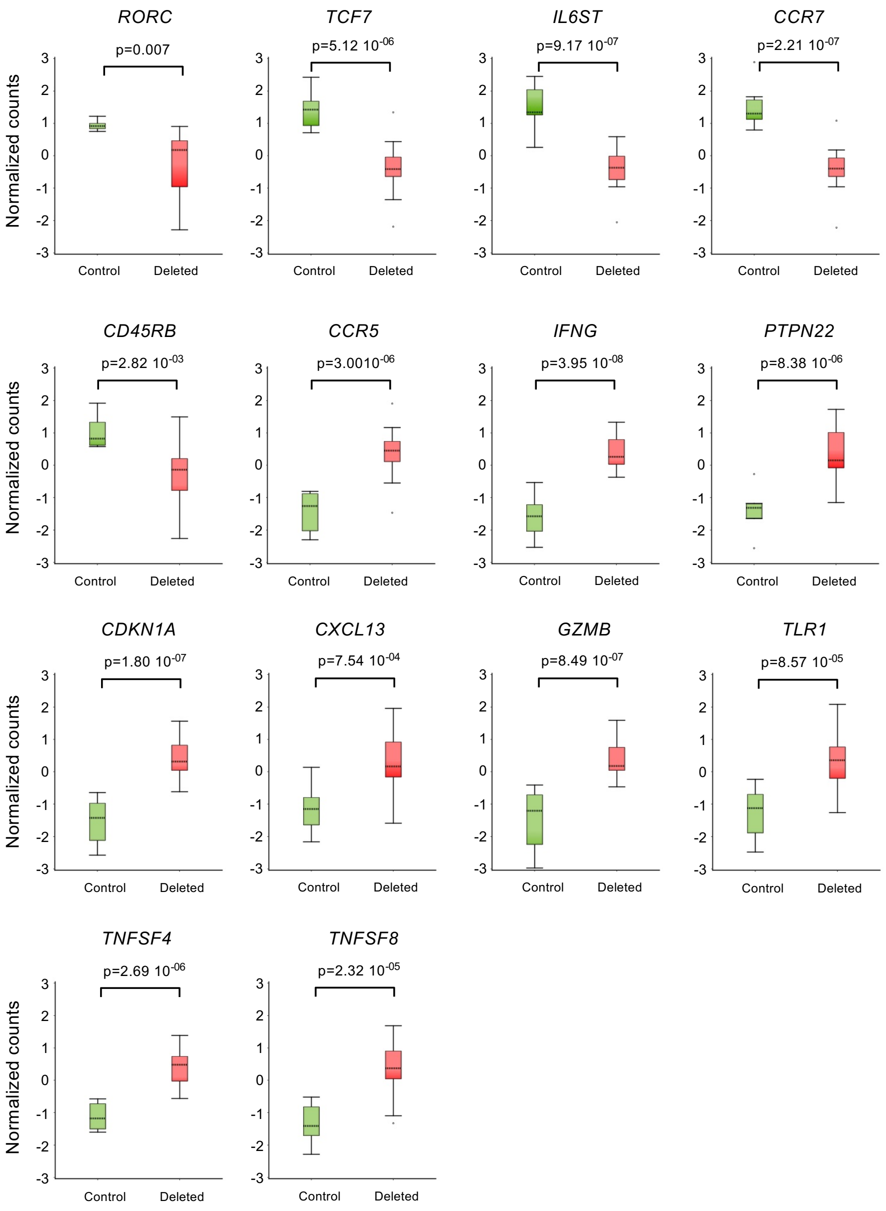

SUPPLEMENTARY FIGURE 7. Engineered deletions of regulatory regions at the *RORC2* locus affect expression of *RORC2* and other immune genes. Box-plot representation of the normalized expression of the 14 genes selected by the ANOVA of Fig. 7F. Two-group comparison was performed in samples carrying genomic deletions at the *RORC* locus (green boxes, 23 samples) and samples transfected with negative control crRNAs (red, 6 samples) from 4 donors. Boxes represent the upper and lower quartiles, the horizontal line is the median, the whiskers are set at the maximum value within the 1.5 IQR. p values for 2-tailed Student T Test are shown. Plots were generated with the Qlucore software. *Source data are provided as a Source Data file.*

Supplementary Table 1. Primers used in chromatin immunoprecipitation assay

RORC -10.9kb	Forward primer	5'- AAACCTCAAAAGCCTCAGCA -3'
	Reverse primer	5'- AATCAGGGAAGGGAGAAGGA -3'
RORC -7.9kb	Forward primer	5'- CATCCCTTCCAGAGTGTTTGC -3'
	Reverse primer	5'- CGTGCCCAGTCCCCTTATAA -3'
RORC -6.4kb	Forward primer	5'- GGTGCCAGGGAGCCTCTT -3'
	Reverse primer	5'- TTGATTAGCTGGGCCTGACTCT -3'
RORCprom	Forward primer	5'- TGTGGTGGGTGCTGTGAATATT -3'
	Reverse primer	5'- GGCCCTTTGCAGGACACTT -3'
RORC -3.9kb	Forward primer	5'- CCACTCATGCTCCCTACTGC -3'
	Reverse primer	5'- AAACACCAGGGCTTCAGAGA -3'
RORC -3.3kb	Forward primer	5'- AGGCTCTGAGGGAAGGA -3'
	Reverse primer	5'- GCCCCAGCCATTTTCTT -3'
RORC -2.5kb	Forward primer	5'- GGCCATGCGTGCCCTTCTGT -3'
	Reverse primer	5'- GTGTGGCCACTCCTACCCTGGA -3'
RORC -1.8kb	Forward primer	5'- GTTGCTTTGCTCAGCATAATCCT -3'
	Reverse primer	5'- TCCCTGACCCGCTTTCCT -3'
RORC2prom	Forward primer	5'- GGCAGACATCCTGTGACTTACCT -3'
	Reverse primer	5'- CTTCCAAGCCTGACCCTCTTT -3'
RORC +0.9kb	Forward primer	5'- AGATGTCTGGGGTTCCTGTG -3'
	Reverse primer	5'- TGTCAGTCCAGGGGTCTTTC -3'
RORC +3kb	Forward primer	5'- GCGGTAAGAGGGAAGTAGGG -3'
	Reverse primer	5'- CTCCCCATCCAGACCAAAT -3'
RORC +3.4kb	Forward primer	5'- CCGTCTTCCCATTCCTAACA -3'
	Reverse primer	5'- CCTTAGCCCTTGGAAAGTCC -3'
RORC +5.5kb	Forward primer	5'- ATCTGAGTTCCCACCACCTG -3'
	Reverse primer	5'- TCCACCCAAACCTCAGAAAG -3'
RORC +6.3kb	Forward primer	5'- AGAAGCAGTGGGTCAAGCAT -3'
	Reverse primer	5'- AAGTAAGCGGGAGGGAGATT -3'
RORC +8.3kb	Forward primer	5'- TCCCTGAGCCTTCACTGCCT -3'
	Reverse primer	5'- CAGCCCTTCGAGTTGAAAAG -3'
RORC +10kb	Forward primer	5'- GGTTGGGCTGTGGTGAGTAT -3'
	Reverse primer	5'- GGTCTGTCCAGTCTGCCATT -3'
CD4prom	Forward primer	5'- TGGGATGTGTGTTGAGTTTGC -3'
	Reverse primer	5'- ATCCGTCTGCCCATTGGA -3'
GAPDHprom	Forward primer	5'- CGGTGCGTGCCCAGTT -3'
	Reverse primer	5'- CCAGTCCCAGCCCAAGGT -3'

Supplementary Table 2. Primers used for genes expression

RORγ	Forward primer	5'- GCAAAGAAGACCCACACCTC -3'
	Reverse primer	5'- GCACCCCTCACAGGTGATAA -3'
RORγt	Forward primer	5'- GCCAAGGCCGGCAGAGCCAA -3’
	Reverse primer	5’- AAGAAGCCCTTGCACCCCTCACA -3’
IFNγ	Forward primer	5'- GCTCTGCATCGTTTTGGGTTCTCTTG -3'
	Reverse primer	5'- CATTCATGTCTTCCTTGATGGTCTCC -3'
TBX21	Forward primer	5'- CAGAATGCCGAGATTACTCAG -3'
	Reverse primer	5' -GGTTGGGTAGGAGAGGAGAG -3'
NFATc1	Forward primer	5'- GCATCACAGGGAAGACCGTGTC -3'
	Reverse primer	5'- GAAGTTCAATGTCGGAGTTTCTGAG -3'
NFATc2	Forward primer	5'- AAACTCGGCTCCAGAATCCA -3'
	Reverse primer	5'- GATGCTGCAGATGGGAATGG -3'
18S	Forward primer	5'- CAGCCACCCGAGAATTATTGAGCA -3'
	Reverse primer	5'- TAGTAGCGACGGGCGGTGTG -3'

Supplementary Table 3. Oligonucleotides for High affinity DNA capture assay. In Red predicted NFAT binding sites. The prosition from *RORC2* TSS is depicted

RORC - 2.5kb	Oligo 1	Forward primer	TTATCTCTGTAGGAAACAGAATGGACAGGG	-2476
		Reverse primer	CCCTGTCCATTCTGTTTCCTACAGAGATAA	
RORC - 1.8Kb	Oligo 2	Forward primer	CCCACGGAAATGCTGAGCAGGGCCCCGGGA GGAAAGCGGGTCAGG	-1905
		Reverse primer	CCTGACCCGCTTTCCTCCCGGGGCCCTGCTC AGCATTTCCGTGGG	
Nucleotides	Oligo 3	Forward primer	ATACCTCCTTATCCCTTTCCCATCCCACCA	-1729
		Reverse primer	TGGTGGGATGGGAAAGGGATAAGGAGGTAT	
RORC2 prom	Oligo 4	Forward primer	TTGGCTCCTAAGGAAATTTCTGGTTTCCTTCT GTG	-580
		Reverse primer	CACAGAAGGAAACCAGAAATTTCCTTAGGAGC CAA	
	Oligo 5	Forward primer	TTACTGCTTCTATCATTTCCATGTATTCCC	-524
		Reverse primer	GGGAATACATGGAAATGATAGAAGCAGTAA	
	Oligo 6	Forward primer	CCTGGCGGGTGGAAACAGCTTTTACCGCGT	-205
		Reverse primer	ACGCGGTAAAAGCTGTTTCCACCCGCCAGG	
	Oligo 7	Forward primer	CATGTGGTTTTGGAATTTTCCAACGCCCCC	-165
		Reverse primer	GGGGGCGTTGGAAAATTCCAAAACCACATG	
Nucleotides	Negative control Oligo	Forward primer	GCACATCAGCTCTCCTCTCCTAGTTTGTGG	-2328
		Reverse primer	CCACAAACTAGGAGAGGAGAGCTGATGTGC	
IL-2 prom	Positive control Oligo	Forward primer	CTAGACACCCCCATATTATTTTTCCAGCATTA	
		Reverse primer	TAATGCTGGAAAAATAATATGGGGGTGTCTAG	
IL-2 prom	Wild type Oligo	Forward primer	CCCCATATTATTTTTCCAGCATTA	
		Reverse primer	TAATGCTGGAAAAATAATATGGGG	
IL-2 prom	Mutated Oligo	Forward primer	CCCCATATTATTCGTCTAGCATTA	
		Reverse primer	TAATGCTAGACGAATAATATGGGG	

Supplementary Table 4. Primers used to generate constructs for reporter gene assay

*RORC2*prom	Forward primer	5’- GCTGCTAGCTCGAGAACCATTTCTGACA -3’
	Reverse primer	5’- CGTTCTGAACCGAGGGACAGGAAC -3’
Inverted *RORC2*prom	Forward primer	5'- GCTAGATCTTCGAGAACCATTTCTGACA -3'
	Reverse primer	5'- TTAGCTAGCCTTGGCTCCCTGTCCTTC -3'
Prom1CNS-2.5/1.8	Forward primer	5'- ATAGGTACCAGCTCTGTGGTCCTGA -3'
	Reverse primer	5'- ATTGCTAGCGTCAGGGGTTAAGGGTA -3'

Supplementary Table 5. Antibodies used for Chromatin Immunoprecipitation Assays

Antibody	Supplier	Reference	Amount used per ChIP sample
Anti-H4ac	Millipore	06-866	5µg
Ant-H3K4me3	Upstate Biotechnology	07-473	2µg
Anti-H3K27me3	Diagenode	C15200181	2µg
Anti-H3K4me1	Millipore	07-436	4µg
Anti-H3K27Ac	Abcam	ab4729	1µg
Anti-NFATc1	Santa cruz	sc-7294	2µg
Anti-NFATc2	Santa cruz	sc-7296	2µg
Anti-NFATc3	Santa cruz	sc-8405	2µg
Anti-p65	Millipore	MAB3026	2µg
Anti-p50	Santa cruz	sc-1190	2µg
Anti-p300	Santa cruz	sc-585	4µg
Anti-p300	Diagenode	C15200211	4µg
Anti-CBP	Diagenode	C15410224	4µg

Supplementary Table 6. crRNA used for CRISPR/cas9 in primary cells

crRNA	Sequence	Strand	Distance from *RORC2* TSS	Number of OFF targets (equal or less than two mismatches)
Upstream O1	TCTGTGGTTAACACATCCTG	+	-2570	0
Downstream O1	GTGCTGAGTGGTCCCTAGAG	-	-2334	2 (2 mismatches in an intron of PPP2R2B, 2 mismatches in an intron of FBLN1)
Upstream O2	CCCAGGAGGAGGTAGCATCC	-	-1991	0
Downstream O2	GGGATAAGGAGGTATAGTCC	-	-1724	0
Upstream O4	CCTCACAAAATACTTTATGT	-	-744	0
Downstream O5	TATATGTGGTGTCTGCAGGG	+	-501	0
Upstream O6	TTCTACTCCTCCTACCCCCG	-	-294	1 (2 mismatches in an intron of TMEM87B)
Downstream O7	TTGTCACTTGGCCACCTGTG	+	-48	0

Supplementary Table 7. Primers used for genotyping CRISPR/Cas9

O1 region	Forward primer	GGCCTCAGGAACTCAGGAAC
	Reverse primer	TCAAGGGACGGCTATGGAGA
O2 region	Forward primer	CACACCATTTCTCCATAGCC
	Reverse primer	GCCAAACAATTCCAGAGACC
O4/5 region	Forward primer	GACACCCTCAAAAGCAGACTTG
	Reverse primer	GAATGGAGCTCACACGAGCC
O6/7region	Forward primer	GGGTATGGCTGTTGGCAGT
	Reverse primer	GATTTGCTCACACTGTTCCCA

Supplementary Table 8. Antibodies used for Imaging Flow Cytometry

Antibody	Supplier	Reference	Amount used per sample
AF647-NFATc2 clone D43B1 XP Rabbit mAb	Cell Signaling Technologies	#5861	0.05 mg / 3.5x10^{6} cells in 100ml
AF647-rabbit IgG isotype control DA1E XP Rabbit mAb	Cell Signaling Technologies	#2985	0.05 mg / 3.5x10^{6} cells in 100ml
anti-NFATc1: clone 7A6	Millipore	MABS409	0.5ug / 3.5x106 cells in 100ml
normal mouse IgG	Santa Cruz Biotechnology	sc-2025	0.5ug / 3.5x106 cells in 100ml
Cy3 conjugated AffiniPure F(ab)2 rabbit anti-mouse	Jackson ImmunoResearch	315-166-045	1:200 dilution
