# Supplementary material for: NFAT primes the human RORC locus for RORγt expression in CD4+ T cells
Source: Nat Commun. 2019 Oct 16;10:4698. doi: 10.1038/s41467-019-12680-x (PMC6795897; doi:10.1038/s41467-019-12680-x)
Supplement: Supplementary file 3 — Reporting Summary [file 41467_2019_12680_MOESM3_ESM.pdf]

nature research

Corresponding author(s): Elisabetta Bianchi

Last updated by author(s): Sep 18, 2019

Reporting Summary

Nature Research wishes to improve the reproducibility of the work that we publish. This form provides structure for consistency and transparency in reporting. For further information on Nature Research policies, see Authors & Referees and the Editorial Policy Checklist.

Statistics

For all statistical analyses, confirm that the following items are present in the figure legend, table legend, main text, or Methods section.

n/a	Confirmed	
☐	☒	The exact sample size (*n*) for each experimental group/condition, given as a discrete number and unit of measurement
☐	☒	A statement on whether measurements were taken from distinct samples or whether the same sample was measured repeatedly
☐	☒	The statistical test(s) used AND whether they are one- or two-sided<br>*Only common tests should be described solely by name; describe more complex techniques in the Methods section.*
☒	☐	A description of all covariates tested
☒	☐	A description of any assumptions or corrections, such as tests of normality and adjustment for multiple comparisons
☐	☒	A full description of the statistical parameters including central tendency (e.g. means) or other basic estimates (e.g. regression coefficient) AND variation (e.g. standard deviation) or associated estimates of uncertainty (e.g. confidence intervals)
☐	☒	For null hypothesis testing, the test statistic (e.g. *F*, *t*, *r*) with confidence intervals, effect sizes, degrees of freedom and *P* value noted<br>*Give P values as exact values whenever suitable.*
☒	☐	For Bayesian analysis, information on the choice of priors and Markov chain Monte Carlo settings
☒	☐	For hierarchical and complex designs, identification of the appropriate level for tests and full reporting of outcomes
☒	☐	Estimates of effect sizes (e.g. Cohen's *d*, Pearson's *r*), indicating how they were calculated

Our web collection on statistics for biologists contains articles on many of the points above.

Software and code

Policy information about availability of computer code

Data collection	Thermo Fisher StepOne Plus qPCR machine, ImageQuant LAS 4000 (Fuji), nCounter technology , ImageStream X MkII Imaging cytometer
Data analysis	Microsoft Excel version 16.16.9, Vista Browser, Prism 8.0.1, nSolver 4.0, Qlucore 3.5, IDEAS software (Amnis), Diva 8, Flowjow10

For manuscripts utilizing custom algorithms or software that are central to the research but not yet described in published literature, software must be made available to editors/reviewers. We strongly encourage code deposition in a community repository (e.g. GitHub). See the Nature Research guidelines for submitting code & software for further information.

Data

Policy information about availability of data

All manuscripts must include a data availability statement. This statement should provide the following information, where applicable:

- Accession codes, unique identifiers, or web links for publicly available datasets
- A list of figures that have associated raw data
- A description of any restrictions on data availability

The datasets generated during and/or analyzed during the current study are available in the Source data file. Raw flow cytometry data are available upon request from authors.

Field-specific reporting

Please select the one below that is the best fit for your research. If you are not sure, read the appropriate sections before making your selection.

☒ Life sciences ☐ Behavioural & social sciences ☐ Ecological, evolutionary & environmental sciences

Life sciences study design

All studies must disclose on these points even when the disclosure is negative.

Sample size	No analysis to predetermine sample size was performed
Data exclusions	From nCounter data, we removed from the analysis: probes with low counts in 100% of the samples (counts below the mean of the negative control probes + 2SD in all samples), probes mapping to multiple genes or probes aligning to polymorphic regions with greater than two SNPs and probes used for housekeeping gene normalization.
Replication	Gene expression, Imaging flow cytometry analysis, Chromatin precipitation and transfection experiments: as indicated as in the corresponding figure legends.<br>Pull-down experiments: at least two experiments were performed and data from replicated experiments are available on request
Randomization	Not applicable to this study
Blinding	Not applicable to this study

Reporting for specific materials, systems and methods

We require information from authors about some types of materials, experimental systems and methods used in many studies. Here, indicate whether each material, system or method listed is relevant to your study. If you are not sure if a list item applies to your research, read the appropriate section before selecting a response.

Materials & experimental systems

n/a	Involved in the study	
☐	☒	Antibodies
☐	☒	Eukaryotic cell lines
☒	☐	Palaeontology
☒	☐	Animals and other organisms
☐	☒	Human research participants
☒	☐	Clinical data

Methods

n/a	Involved in the study	
☒	☐	ChIP-seq
☐	☒	Flow cytometry
☒	☐	MRI-based neuroimaging

Antibodies

Antibodies used	Please see supplementary table 1
Validation	Antibodies for ChIP experiments and for imaging were validated by the suppliers indicated in supplementary table 1.<br>ChIP experiments were validated by comparison to an irrelevant antibody.

Eukaryotic cell lines

Policy information about cell lines

Cell line source(s)	American Type Culture Collection
Authentication	No further authentication was performed
Mycoplasma contamination	Cell lines were tested negative for mycoplasma contamination
Commonly misidentified lines<br>(See ICLAC register)	*Name any commonly misidentified cell lines used in the study and provide a rationale for their use.*

Human research participants

Policy information about studies involving human research participants

Population characteristics	• Biospecimen type 1) T cells from adult peripheral blood were isolated from buffy-coats (Hôpital Saint Louis, Paris, France).<br>Disease status : healthy donors.<br>Collection mechanism, stabilization, preservation : heparinized buffy coats were delivered to the laboratory within 4 hours of collection and immediately processed for analysis.<br><br>• Biospecimen type 2) Cord Blood was obtained from the AP-HP Cord Blood Bank (Hôpital Saint Louis, Paris, France), as units

discarded following screening for allogeneic transplantation. Disease status : healthy donors
Collection mechanism, stabilization, preservation :heparinized cord blood samples were delivered to the laboratory and immediately processed for analysis. Only specimens delivered within 12 hours from delivery were processed.

• Biospecimen type 3) thymic fragments. Human thymi were removed during cardiac surgery at the Pediatric Cardiac Surgery Department of Hôpital Necker, Paris, France. Disease status : anonymous pediatric donors undergoing heart surgery. No clinical diagnosis available.
Collection mechanism, stabilization, preservation : thymic fragments removed during surgery were immediately stored in PBS at room temperature, and delivered to the laboratory within one hour from removal. Samples were processed on ice for cell separation and paraformaldehyde fixation before further sorting of populations or sonication. Sonicated samples were stored overnight at -80°C before processing for chromatin immunoprecipitation.

Clinical characteristics of donors/patients : Not available.
Vital state of donors/patients : Alive
Storage temperature : fixed and sonicated samples for chromatin immunoprecipitation were stored overnight at -80°C. All other samples were immediately processed.
Type of long-term preservation : no long term preservation.
Storage duration : Blood and thymic samples were used the day of acquisition.
Shipping temperature : samples were kept at 20-25°C during shipment.
Composition assessment and selection : all specimens were tested for a panel of infectious agents (including HBV, HCV, HIV, toxoplasma). Only not infected samples were used.

Recruitment

Not applicable

Ethics oversight

Research protocols were approved by the Institut Pasteur, and regional ethical committees as detailed in material and methods section.

Note that full information on the approval of the study protocol must also be provided in the manuscript.

Flow Cytometry

Plots

Confirm that:

☒ The axis labels state the marker and fluorochrome used (e.g. CD4-FITC).

☒ The axis scales are clearly visible. Include numbers along axes only for bottom left plot of group (a 'group' is an analysis of identical markers).

☐ All plots are contour plots with outliers or pseudocolor plots.

☒ A numerical value for number of cells or percentage (with statistics) is provided.

Methodology

Sample preparation

Human CD4+ T cells were isolated by density gradient centrifugation and magnetic cell sorting.

Instrument

BD FACS ARIA III, ImageStream X MkII Imaging cytometer.

Software

IDEAS software (Amnis), Diva 8, Flowjow10

Cell population abundance

Not applicable

Gating strategy

1. Lymphocyte population and single cells (FSC, SSC).
2. DAPI+ cells for ImageStream analysis. DAPI- Atto550 high for sorting for CRISPR/Cas9 experiments.

☒ Tick this box to confirm that a figure exemplifying the gating strategy is provided in the Supplementary Information.
